# Correction: Essential requirement of cytochrome c release for caspase activation by procaspase-activating compound defined by cellular models

**DOI:** 10.1038/s41419-025-08119-5

**Published:** 2025-10-21

**Authors:** M. Seervi, J. Joseph, P. K. Sobhan, B. C. Bhavya, T. R. Santhoshkumar

**Affiliations:** Integrated Cancer Research Program, Rajiv Gandhi Centre for Biotechnology, Thiruvananthapuram, India

Correction to: *Cell Death and Disease* 10.1038/cddis.2011.90, published online 08 September 2011

We noted an error in the figure 3a of the manuscript that shows nuclear condensation image of MCF7 C3 cells treated with PAC-1 for 48h. During the image compilation, a part of PAC1- 36h treatment image (figure 5a) was mistakenly cropped and presented as a PAC-1 treatment for 48h in Fig 3a (highlighted in attached original data Tiff file). We captured multiple images of chromatin condensation induced by PAC-1 in the cells at various time points but unfortunately, an error happened during the panel arrangement.

We kindly request this error correction in our article by inserting the right Hoechst image (PAC1, 48h for MCF C3 as shown in the original data Tiff file). The amended file for Fig 3 is also provided after inserting this correction (Amended Fig. 3 Tiff file).

**Original Fig. 3.** 
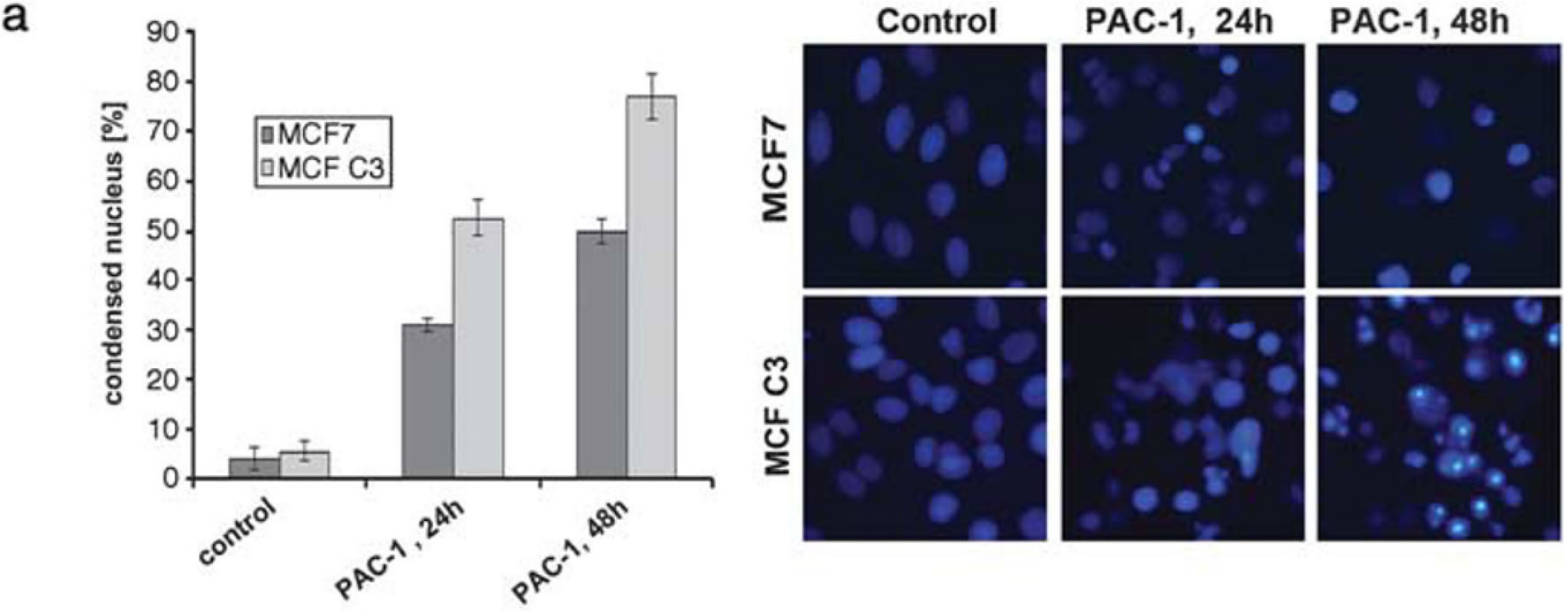


**Amended Fig. 3.** 
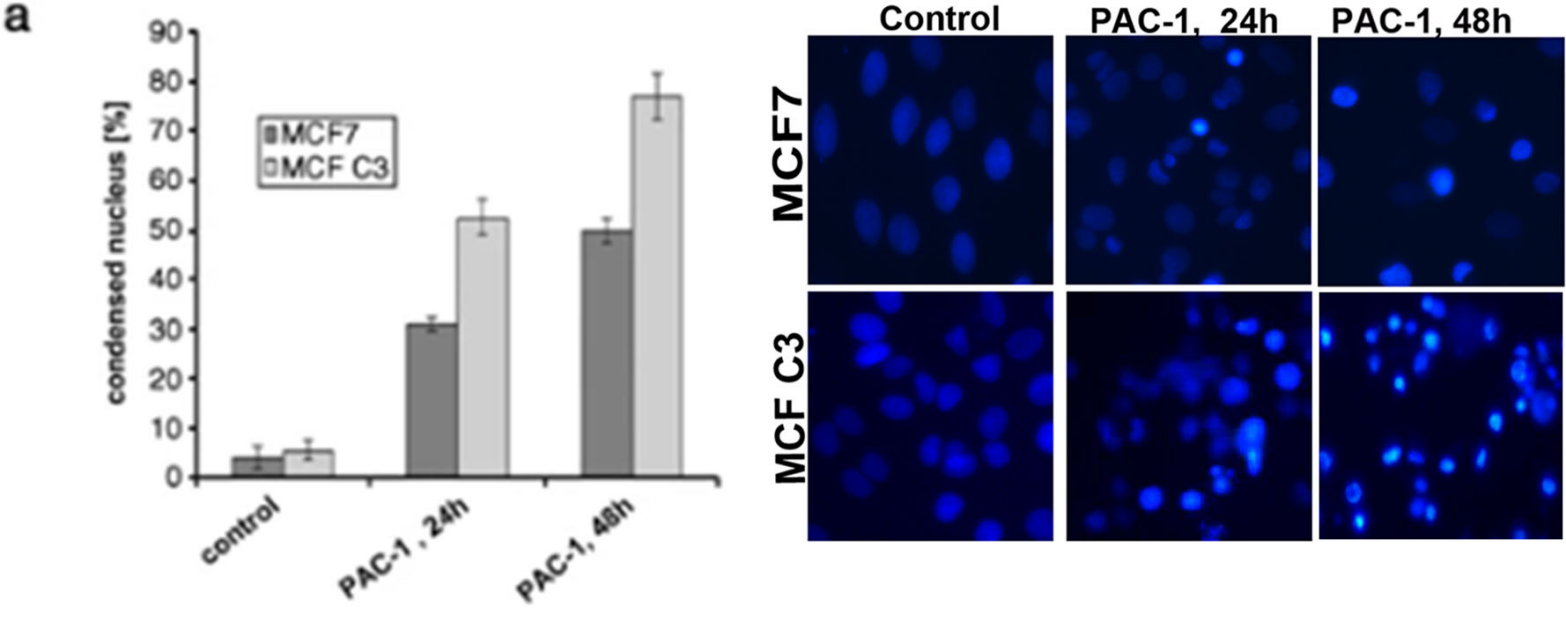


Kindly note that, the images are only representation of quantitative graph and does not in any way affect the accuracy of the article or invalidate the conclusion derived. It was a genuine error from our side while compiling.

The work was initiated almost 20 years back and during the period of 2006-2010, we were having only one inverted fluorescence microscope that was controlled by IP Lab software (Scananalytic Inc., currently not available and discontinued from 2010). This equipment was used by many investigators. So, after Hoechst staining, we used to count the cells with condensed nuclei and representative images were taken for presentation. Among this, few images are copied for presentation in PowerPoint software as we were having only one IP lab software licence. Later, while images are assembled for publication, relevant fields are cropped from the images of cells treated with the PAC1 at respective hours. In this case, it appears that while copying 48h of images, again another area from the 36h of the image was cropped by mistake possibly due to lack of visible condensation difference between these time point, and same cell with same drug concentration. We deeply regret for this error happened during the figure panel preparation and sincerely apologise.

Please note that with this correction, none of the finding of the work are affected or changed.

## Supplementary information

Original Data (Fig 3a -2011)(1)

