# Supplementary figures and images for: Correction: Essential requirement of cytochrome c release for caspase activation by procaspase-activating compound defined by cellular models
Source: Cell Death Dis. 2025 Oct 21;16(1):748. doi: 10.1038/s41419-025-08119-5 (PMC12540989; doi:10.1038/s41419-025-08119-5)

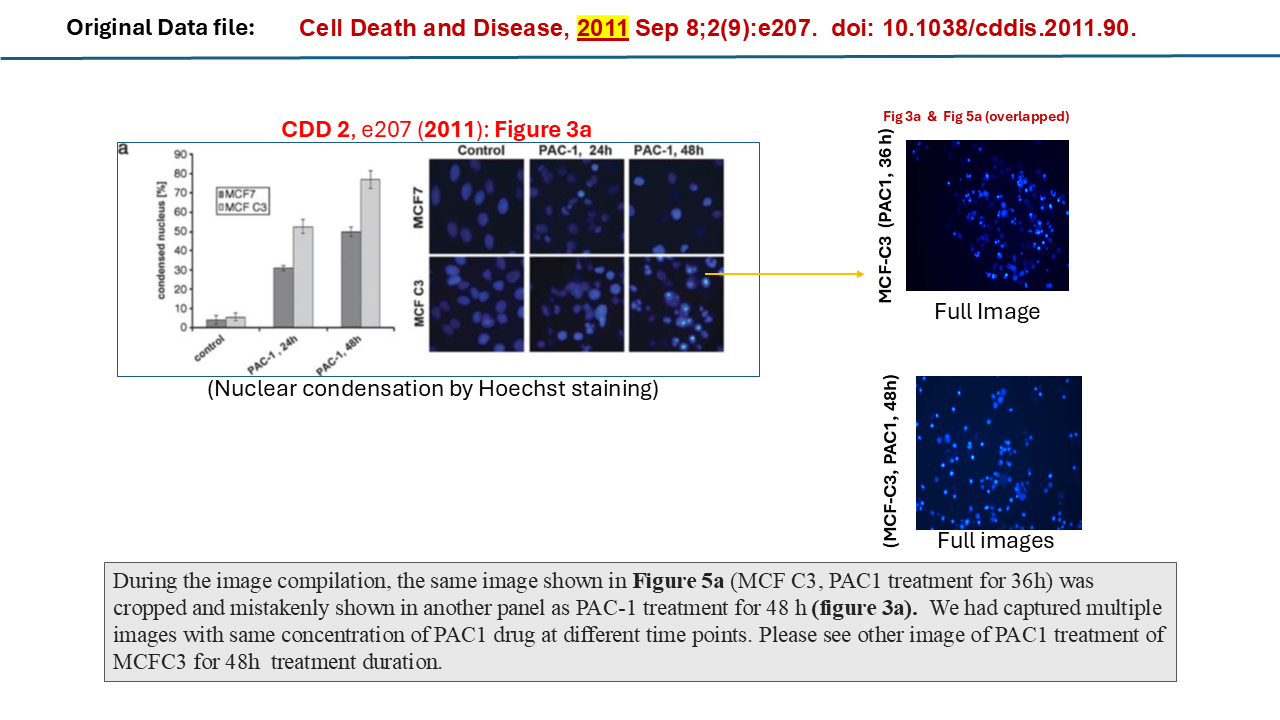

Supplement: Supplementary file 1 — Original Data (Fig 3a -2011)(1) [file 41419_2025_8119_MOESM1_ESM.tiff]
